# Potential of phage depolymerase for the treatment of bacterial biofilms

**DOI:** 10.1080/21505594.2023.2273567

**Published:** 2023-10-31

**Authors:** Zhimin Guo, Mengmeng Liu, Dan Zhang

**Affiliations:** a Department of Laboratory Medicine, Infectious Diseases and Pathogen Biology Center, The First Hospital of Jilin University, Changchun, China; b Department of Laboratory Medicine, The First Hospital of Jilin University, Changchun, China; c Department of Hepatological Surgery, The First Hospital of Jilin University, Changchun, China

**Keywords:** Phage, depolymerase, bacteria, biofilm, polysaccharide, treatment

## Abstract

Resistance of bacteria to antibiotics is a major concern in medicine and veterinary science. The bacterial biofilm structures not only prevent the penetration of drugs into cells within the biofilm’s interior but also aid in evasion of the host immune system. Hence, there is an urgent need to develop novel therapeutic approaches against bacterial biofilms. One potential strategy to counter biofilms is to use phage depolymerases that degrade the matrix structure of the bacteria and enable access to bacterial cells. This review mainly discusses the methods by which phage depolymerases enhance the efficacy of the human immune system and the therapeutic applications of some phage depolymerases, such as single phage depolymerase application, combined therapy with phage depolymerase and antibiotics, and phage depolymerase cocktails, for treating bacterial biofilms. This review also summarizes the relationship between bacterial biofilms and antibiotic resistance.

## Introduction

It is estimated that antimicrobial-resistant bacterial infections will become the leading cause of death by 2050 [1]. The formation of bacterial biofilms is considered to be one of the major causes of antibiotic resistance in bacteria [2]. Bacterial biofilm acts as a virulence factor. Biofilms can prevent the penetration of drugs through their matrix and reduce the ability of antibiotics to reach the surface of bacterial cells. Furthermore, biofilms enable bacteria to evade the host immune system [3]. Some pathogenic bacteria, such as *E. coli*, *K. pneumoniae*, *P. aeruginosa*, *S. aureus*, and *E. faecalis*, can form biofilms on the surfaces of medical instruments and human and animal tissues [4]. In fact, it has been estimated that bacterial biofilms are related to approximately 80% of chronic and recurrent bacterial infections in humans, including cystic fibrosis, endocarditis, meningitis, osteomyelitis, rhinosinusitis, and periodontitis, as well as kidney and prosthesis infections [5]. Bacteria that are susceptible to antibiotics when in planktonic form can develop increased antibiotic resistance after biofilm formation on a suitable surface [6]. The possible reasons may be bacterial biofilms reducing the penetration of antibiotics into the deeper layers, biofilm bacteria growing slowly in the deeper layers or biofilm bacteria developing molecular mechanisms of antibiotic resistance. There is, thus, an urgent need to develop novel therapeutic approaches, besides the conventional antibiotics used against bacterial biofilms.

The use of phage depolymerases is a promising therapeutic strategy for preventing and controlling bacterial biofilm-associated infections [7]. Some phages have developed the ability to degrade the polysaccharide-based structures produced by biofilm-forming bacteria to gain entry into bacterial cells and replicate their genetic information [8,9]. Not all phages can encode depolymerases with exopolysaccharide-degrading activity. The depolymerases are encoded by some phages that infect the encapsulated bacteria. Phage depolymerases are encoded in the same open reading frames as phage structural proteins or in close proximity to these genes, which are located mainly on tail fibres, base plates, and neck [10]. Phage depolymerases can be divided into two main groups: hydrolases (EC 3.2.1.-) and lyases (EC 4.2.2.-), based on the degradation mode of the carbohydrate polymers on the surface of the bacteria [11]. These depolymerases can degrade polysaccharides by recognizing specific ligands on the bacterial surface. The specific binding to capsular polysaccharides (CPSs) and lipopolysaccharides (LPSs) results in the destruction of repeating units of the polysaccharide [12]. Phage depolymerases do not directly kill the bacteria; instead, they strip the protective polysaccharide layers from the bacterial cells, which exposes and sensitizes them to components of the immune system or to antibacterial agents. Moreover, the degradation of polysaccharides by phage depolymerases can increase the penetration of antibiotics into the biofilm. Thus, phage depolymerases demonstrate a synergistic effect with some antibiotics against biofilm-forming pathogens [13–16]. In the treatment of bacterial infections, phage depolymerase is compared with phage endolysin and phage holin in Table 1.

**Table 1.** Comparison of depolymerase, endolysin and holin derived from phages in the treatment of bacterial infections.

Aspect	Depolymerase	Endolysin	Holin
Belongs to	a part of phage	almost all phages	almost all phages
Action site	polysaccharide	peptidoglycan	membrane
Specificity	narrow spectrum: one or limited to a bacterial species	narrow spectrum: one or limited to a bacterial species	broad spectrum: Gram-positive or Gram-negative species or both
Mechanism	degrade polysaccharide of bacterial biofilm	lysis cell wall of bacteria	punch bacterial membrane
Evolution	co-evolve with bacteria	co-evolve with bacteria	co-evolve with bacteria
Expression process	easy	easy	difficult
Interaction with bacteria	non-bacteriostatic and non-bacteriacidal	bacteriacidal	bacteriacidal
Affect of microbiota	No	No	Yes

This review discusses the enhancing effect of phage depolymerase on the human immune system and the therapeutic applications of some phage depolymerases, such as single phage depolymerase application, combination therapy with phage depolymerase and antibiotics, and phage depolymerase cocktails, for the treatment of bacterial biofilms. Additionally, the relationships between bacterial biofilms and antibiotic resistance have been examined.

## Relationship between bacterial biofilms and antibiotic resistence

Bacterial biofilms are multicellular communities that consist of water (up to 97%), microbial cells (2%–5%), exopolysaccharides (EPSs) (1%–2%), proteins (<1%–2%), and DNA and RNA (<1%–2%) [17]. Biofilms maintain bacterial structural integrity, help them to adhere to biotic and abiotic surfaces, and protect them against antibiotics or the host’s immune system.

Conventional antibiotics can kill growing and dividing bacterial cells, such as planktonic bacteria, that is, those in free-living form [18]. However, the minimum inhibitory concentrations (MICs) of conventional antibiotics for biofilm bacteria are 100–1000 times higher than those for planktonic bacteria [19].

### Formation of the bacterial biofilm

Biofilm formation can be influenced by numerous factors, such as the condition of the surface on which the biofilm form is formed, cellular structures, and chemical and physical growth factors [20]. Hence, the formation of bacterial biofilm is a complex process requiring quorum sensing and different sets of genes for transcription and translation.

**Figure 1.**
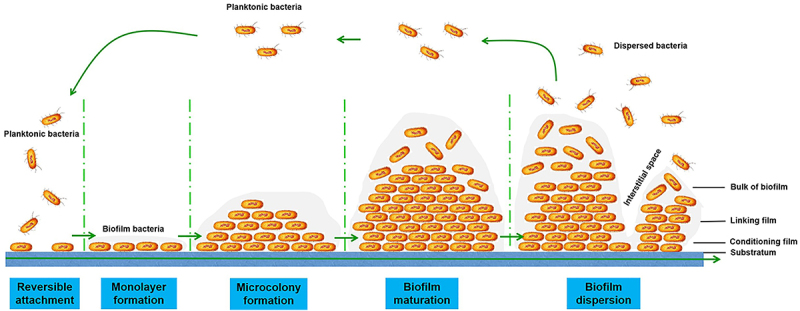
Schematic overview of bacterial biofilm formation and development stages. Biofilm formation starts with reversible adhesion of planktonic cells, and then with irreversible adhesion to the surface. The microbial cells continue to multiply and form micro-colonies and eventually develop into the mature biofilm. In the last stage, biofilm bacteria detach from the mature biofilm and disperse as planktonic state [21–23].

Bacterial biofilm formation can be classified into five stages (Figure 1). The first stage is reversible attachment, wherein the cells newly and loosely attach to the surface via electrostatic forces, van der Waals forces, and hydrophobic interactions [24]. At this stage the attachment is very weak and reversible. The bacteria either commit to the biofilm mode or return to the planktonic lifestyle. Fimbriae, pili, and flagella contribute to their attachment to rough and hydrophobic substances. The second stage of bacterial biofilm formation is monolayer formation, wherein the loose bacteria begin to produce extracellular polymeric substances and consolidate the attachment process [21]. After monolayer formation, irreversible adhesion occurs. The third stage is microcolony formation, wherein various microbial cells continue to accumulate and grow into multilayered cell clusters surrounding EPS, thus leading to the formation of microcolonies and three-dimensional structures [22,24]. In the fourth stage, the microcolonies develop into mature biofilms that may be mushroom- or tower-like in shape with fluid-filled channels. These channels ensure the diffusion and circulation of nutrients, oxygen, and essential substances within the microenvironment [23]. According to Marchabas, matured biofilm structure includes bulk of biofilm, linking film, conditioning film, and the substratum from the outside to the inside [25]. Biofilm dispersal, the last stage of biofilm formation, involves detachment of the bacteria from the mature biofilm and their conversion into a planktonic state. This is a cyclical process, with the detached bacterial cells potentially being able to colonize new surfaces [24]. Different saccharolytic enzymes, such as N-acetyl-heparosan lyase from *E. coli* [26], alginate lyase from *P. aeruginosa* [27], beta-N-acetyl-glucosaminidase (DspB) from *A. actinomycetemcomitans* [28], and hyaluronidase from *Streptococcus equi* [29], are produced during the final stage to facilitate bacterial release from the biofilm and the modulation of biofilm structure.

### Bacterial biofilms reduce the penetration of antibiotics into deeper layers

Bacterial biofilm is a complex three-dimensional structure comprising bacteria, EPSs, metabolites, and nutrients. EPS forms the skeleton of the biofilm [30], which maintains the integrity and persistence of the biofilm architecture. EPS can prevent or retard the penetration of antibiotics into the deeper layers of the biofilm independently or in combination with eDNAs [5].

The ability of antibiotics to penetrate a bacterial biofilm varies among the various classes of antibiotics and bacterial genera. Singh *et al.* [31] found that the average percentage reductions in the penetration of vancomycin, chloramphenicol, amikacin, ciprofloxacin, imipenem, cefotaxime, and tetracycline were 57%, 34%, 22%, 18%, 14%, 11%, and 9%, respectively, in *S. epidermidis*, *S. aureus*, *K. pneumoniae*, and *E. coli* biofilms. In another study, polysaccharide intercellular adhesin (PIA) was shown to reduce the penetration of many antibiotics, such as oxacillin, cefotaxime, teicoplanin, and vancomycin, via the biofilm [32]. PIA, also known as poly-β-1–6-N-acetylglucosamine (PNAG), is the only EPS produced by *staphylococci* [33]. PIA determines the cell surface hydrophobicity of *S. epidermidis* and mediates the initial adherence of the biofilms to some extent [34]. Different bacterial strains might have different types of EPSs, and while some bacteria produce several different types, others produce only one dominant EPS molecule. Anderl *et al* [35]. found that ampicillin did not penetrate the biofilm of *K. pneumoniae*, which indicates the resistance of this species to ampicillin when in biofilm form. Hoyle *et al* [36]. reported a decrease in the diffusion of piperacillin through *P. aeruginosa* biofilms formed on dialysis membranes. *P. aeruginosa* biofilms prevented the entry of antibiotics into bacterial cells, and imipenem or ceftazidime at a concentration of 2560 μg/mL could not eradicate these biofilms. It was also reported that the permeation rates of macrolides, fluoroquinolones, beta-lactams, gentamicin, and amikacin through the alginate of *P. aeruginosa* strain 214 were reported to be 100%, >75%, >75%, 73%, and 59%, respectively, which indicates that biofilms can limit the permeation rates of antibiotics to various degrees [37].

### Biofilm bacteria grow slowly in deeper layers

The bacteria in the deeper layers of biofilms lack oxygen, as reported by Wu *et al* [38]. In one study, the oxygen concentration within the gel biofilm was measured using microelectrodes. A decrease in the oxygen concentration to < 3% of air saturation at a depth of 500 μm was reported in *S. aureus* biofilm [39]. Elsewhere [40], it was documented that the strains exhibited low oxidoreductase activities, which included reductions in the expression levels of pyruvate dehydrogenase, ethanol dehydrogenase, glycerol-3-phosphate dehydrogenase, succinate dehydrogenase, and cytochromes bo and aa3 under anaerobic conditions. In another study on a *P. aeruginosa* clinical isolate, bottom-up proteomic analysis showed that the levels of L-arginine and polyamine metabolism were higher in anoxic regions of biofilms than in oxic ones [41].

Besides oxygen limitations, biofilms also suffer from nutrient limitations that affect bacterial growth. Several nutrients exert multiple effects on the metabolism of the biofilm bacteria, with L-arginine being particularly prominent. Indeed, arginine and aspartic acid were reported to exert opposite effects on biofilm formation in *P. putida* KT2440 and Rup4959 [42]. Similarly, Mills *et al* [43]. observed that low concentrations of L-arginine induced an increase in the concentration of c-di-GMP in *Salmonella typhimurium*. Thus, L-arginine may be used to cope with biofilms based on the nutrient defining solution. Anderl *et al* [44]. found that bacteria in stationary phase could be protected from antibiotics in the absence of carbon and nitrogen within the culture medium. The pH of the biofilm is a key factor determining bacterial metabolism and ranges from 5.6 in deeper layers of biofilms to 7.0 in superficial layers [45]. Low pH can directly reduce the activities of oxacillin [46]. A low nutrient level influences the metabolic state of biofilm bacteria.

These slow-growing bacteria deep in biofilms are more resistant to antimicrobial agents than those growing at an intermediate rate [47]. For example, Zheng and Stewart [48] noted that rifampin penetrates the biofilm of *S. epidermidis* but does not kill these bacteria. The reason for this failure to achieve bacterial killing was not the inadequate penetration of rifampin through the biofilm, but the slow or absent bacterial growth. Results showed that the average growth rate of biofilm bacteria was 0.035 ± 0.004 h^−1^, whereas the average growth rates of the stationary-phase and exponential-phase bacteria were 0.15 ± 0.06 h^−1^ and 0.82 ± 0.34 h^−1^, respectively. Other studies also showed that the slow growth of biofilm bacteria enable them to evade the effect of rifampicin [49,50]. These studies establish that metabolically inactive bacteria can escape the effects of conventional antibiotics.

### Biofilm bacteria develop molecular mechanisms of antibiotic resistance

Biofilms provide an the ideal environment for horizontal gene transfer and the development of multidrug resistance [51]. Conjugation is the most common mechanism by which the horizontal transfer of resistance genes occurs within biofilms. Indeed, a study reported that the transfer rate of the conjugative plasmid pGO1 in *S. aureus* biofilms was nearly 16,000 times higher than that observed in the planktonic state [52]. Moreover, Kouzel *et al* [53]. quantified the acquisition and spread of multidrug resistance and observed that the transfer efficiencies of *ermC* and *aadA* were higher during the early stages of *N. gonorrhoeae* biofilm formation. Resistance genes can also be transferred between different bacterial species in biofilms. For instance, an *in vitro* biofilm experiment demonstrated the transfer of the blaNDM-1 gene from *Enterobacteriaceae* into *P. aeruginosa* and *A. baumannii* via conjugation [54]. Transduction, which occurs via temperate phages, is another mechanism by which resistance genes can be exchanged between bacteria. ϕ731 is a Shiga-toxin-encoding phage with a chloramphenicol resistance gene, which was reported to be transferred to pathogenic *E. coli* in biofilms at both 20°C and 37°C. It was also found that *S. epidermidis* temperate phages could spread antimicrobial resistance genes via transduction [55]. Transformation in the biofilm is the third mechanism of horizontal gene transfer. For example, *S. pneumoniae* can acquire genes conferring resistance to streptomycin or trimethoprim resistance genes by recombination via the transformation of DNA from the environment. Two pneumococcal strains, S2^Tet^ and S4^Str^, were incubated together, and the emergence of double-resistant pneumococci was observed at a recombination frequency of 2.5 × 10^−4^ 4 h post-inoculation [56].

In addition to gene transfer, gene mutations in biofilm bacteria can lead to resistance. The spontaneous mutation rates of several bacteria were reported to range from approximately 10^−10^ to 10^−9^ per nucleotide per generation [57]. Furthermore, in biofilms, the occurrence rates of mutations are higher than the spontaneous mutation rates. This kind of mutation is usually called high mutability or hypermutability. In biofilm bacteria, mutations can confer an evolutionary advantage, especially under the pressure of nonlethal-dose antibiotics and growth restrictions. The presence of bacteria with hypermutability can lead to antibiotic resistance. *P. aeruginosa* is one of the most prone to producing biofilms bacteria. Thus, its hypermutation is considered to be the main driver for the development of antimicrobial resistance in this species in patients with chronic infections. One study reported a 105-fold increase in mutation frequency in biofilm bacteria compared with that in planktonic bacteria [58]. Furthermore, several enzymes that protect DNA from oxidative damage were found to be downregulated in *P. aeruginosa* biofilms. For instance, the major pseudomonal antioxidant catalase encoded by *katA* was found to be downregulated by 7.7 folds. The downregulation of antioxidant enzymes leads to the accumulation of DNA damage, which accelerates the rate of mutagenic events [58]. Sultan *et al*. [59] observed the coexistence of *qnrA*, *qnrB*, *qnrS*, and *gyrA* gene mutations and biofilm production in almost 40% of quinolone-resistant uropathogenic *E. coli*. These findings indicate the relationship between gene mutations and biofilm production.

It is worth mentioning that some researchers believe that efflux pumps are not associated with biofilm formation. For example, Türkel *et al* [60]. explored the relationship between efflux pump-associated gene expression levels and biofilm formation by collecting 100 clinical extended-spectrum β-lactamase-producing *K. pneumoniae* isolates and examining their biofilm-forming capabilities [60]. The expression levels of *AcrA*, *ketM*, *kdeA*, *kpnEF*, and *kexD*, which are related to efflux pumps, were measured. Interestingly, no correlations were observed between the expression levels of these efflux pump genes and biofilm formation. In another study, Knight *et al*. reported that mutation of the major efflux pump gene Δ*adeJ* led to a minor decrease in biofilm formation [61]. Moreover, Li *et al* [62]. documented significant correlations between the efflux pump MexAB-OprM phenotype and biofilm formation in 110 carbapenem-resistant *P. aeruginosa* strains. Thus, there is a need for additional studies to determine the relationship between efflux pump genes and biofilm formation.

## Applications of phage depolymerase for the treating of bacterial biofilms

**Figure 2.**
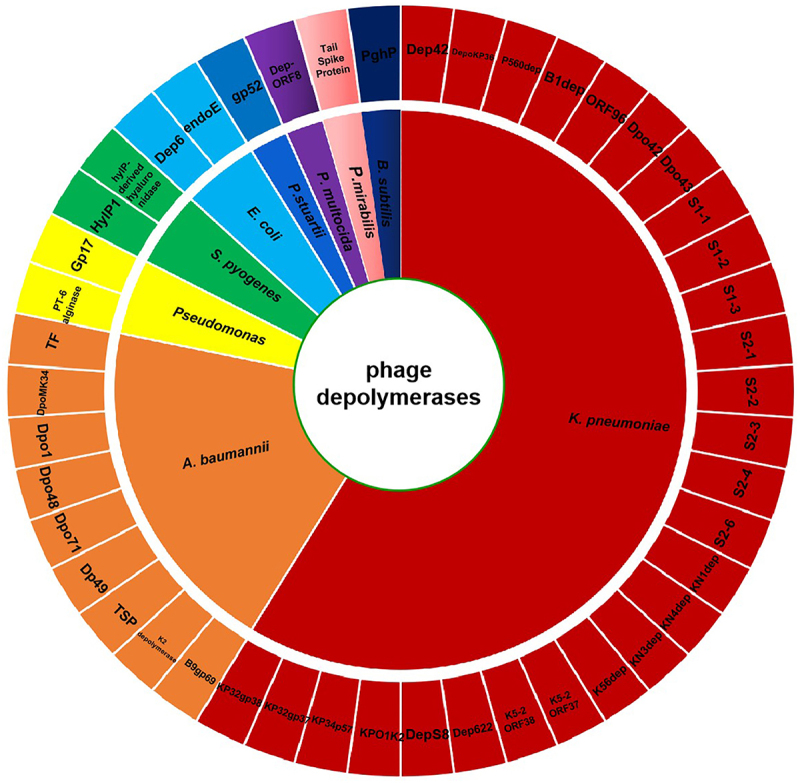
Wheel diagram summary depicting the classification of phage depolymerases reported in the treatment of bacterial biofilm and their corresponding bacterium genus targets.

Antibiotic treatment often fails in clinical medicine owing to the impermeability of bacterial biofilms. Thus, other methods, such as the use of phage depolymerases, have been attempted to counter the resistance conferred by such biofilms. Phages are natural predators of bacteria, but not all phages can encode depolymerases with exopolysaccharide-degrading activity. Depolymerases are encoded by some phages that infect the encapsulated bacteria, such as *E. coli* K1 [63], *E. coli* K20 [64], *K. pneumoniae* K22 [65], *K. pneumoniae* K23 [66], *K. pneumoniae* K64 [67], *A. baumannii* K26 [68], and *A. baumannii* K92 [69]. Although the use of a single phage depolymerase can combat bacterial biofilms, the complete eradication of bacterial biofilms may require the application of multi-phage depolymerase cocktails or their combined use with antibiotics. An overall classification of phage depolymerases reported in the treatment of bacterial biofilm and their corresponding bacterium genus targets are summarized in Figure 2.

**Figure 3.**
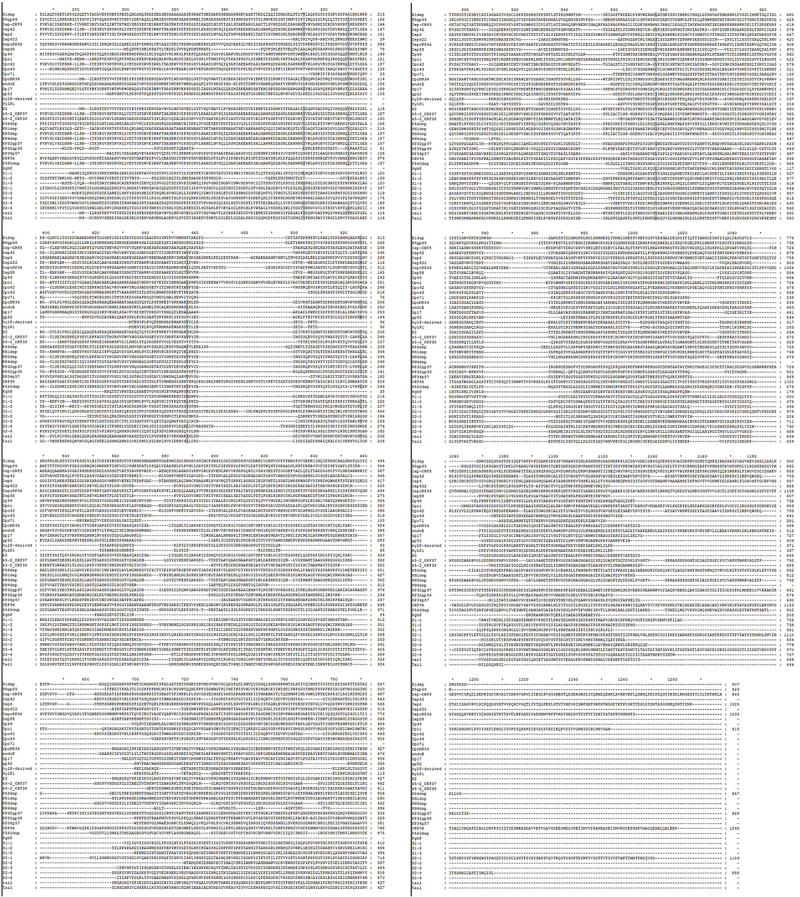
The protein sequence homologies of phage depolymerases.

The structure of phage depolymerase may be differ among different species of depolymerases. Whereas, phage depolymerase is a trimer crystal state in general [70,71]. According to the crystal structure of KP32gp38 and K1 CPS depolymerase, each monomer has three distinct domains: the N-terminal particle-binding domain, the central receptor-binding domain, the C-terminal β sandwich domain. The N-terminal domain consists of β-sheet and α-helix. Three monomers fold into a barrel-like structure. The central receptor-binding domain mainly features β-helix and contains at least one distinct carbohydrate‑binding site. The C-terminal β sandwich domain has a lectin-like fold formed by β strands. Three parallel chains tightly packed together to form a highly stable screw-like trimeric structure [70,71]. The protein sequences of phage depolymerases are compared in Figure 3.

### Application of a single phage depolymerase

To eliminate polysaccharide-producing bacteria, phages have evolved several species of depolymerases to overcome the capsular structure surrounding the bacteria. Phage depolymerases can be categorized into two main groups, namely, hydrolases (EC 3.2.1.-) and lyases (EC 4.2.2.-), based on the mode of degradation of carbohydrate polymers on the bacterial surface [11]. The hydrolases include sialidases (EC 3.2.1.18) [72], poly-γ-glutamate (γ-PGA) hydrolases (EC 3.4.19.9) [73], xylosidases (EC 3.2.1.37) [74], levanases (EC 3.2.1.65) [75], dextranases (EC 3.2.1.11) [76], lipases (EC 3.1.1.3) [77], and rhamnosidases (EC 3.2.1.40) [10]. Hydrolases are a group of enzymes that catalyse the cleavage of 1,4- glycosidic bonds in the glycosidic linkage, thereby degrading CPSs, EPSs, LPSs, and O-polysaccharides. In contrast, lyases cleave the glycosidic linkage between monosaccharides and the C4 position in uronic acid and introduce a double bond via the β-elimination mechanism between the C4 and C5 positions in nonreducing uronic acid. Some phage depolymerases are listed in Table 2 according to those with defined classes and those yet to be defined.

**Table 2.** Therapy of phage depolymerase in the treatment of bacterial biofilm.

Depolymerase	Enzyme class (EC Number)	Bacterium genus	Origin phage	Application model	Mechanism	Substrate	Study references
Endosialidase E (endoE)	sialidases or neuraminidases (EC 3.2.1.18)	*E. coli* K1	phage A192PP	neonatal rat bacteraemia and meningitis model	hydrolyzes α-2,8-linked sialic acid	sialic acid	[72,78]
PghP	poly-γ-glutamate (γ-PGA) hydrolase (EC 3.4.19.9)	*Bacillus subtilis*	PhiNIT1/ΦNIT1	In vitro	randomly hydrolyzes γ-PGA into oligo-γ-glutamates and specifically into tri-, tetra-,and penta-γ-glutamate.	γ-PGA	[73]
HylP1	hyaluronate lyases (4.2.99.1)	*S. pyogenes*	SF37.1 prophage	In vitro	catalyze a β-elimination reaction	hyaluronan (HA)	[79]
hylP-derived hyaluronidase	hyaluronate lyases (4.2.99.1)	S. pyogenes	phage H4489A	In vitro	cleave N-acetylglucosaminidic bonds of HA	hyaluronan (HA)	[80]
PT-6 alginase	Alginate lyases (EC 4.2.2)	*Pseudomonas aeruginosa*	PT-6	In vitro	Degradate alginic acids	alginic acids	[81]
Gp17	pectate lyase (EC 4.2.2.2)	*Pseudomonas putida*	phages φ15	In vitro	degradation of galacturonic acids	galacturonic acid	[82]
Dpo48	pectate lyase (EC 4.2.2.2)	*A.baumannii*	phage IME200	In vitro	degradation of galacturonic acids	galacturonic acid	[83]
Tail Spike Protein	pectate lyase (EC 4.2.2.2)	*Proteus mirabilis*	vB_PmiS_PM-CJR	Galleria mellonella larvae	degrade *Proteus* biofilm	biofilm	[84]
tailspike protein (TSP)	Undefined	*A.baumannii*	ϕAB6 phage	zebrafish	degrade *A. baumannii* biofilm	biofilm	[7]
Dp49	Undefined	*A.baumannii*	vB_AbaM_IME285	mice	degrade *A. baumannii* biofilm	biofilm	[85]
Dpo71	Undefined	*A.baumannii*	IME-AB2	Galleria mellonella	inhibit biofilm formation, disrupt the preformed biofilm and enhance the bactericidal effect of colistin	biofilm	[16]
gp52	Undefined	*Providencia stuartii*	vB_PstP_Stuart	In vitro	Degrade exopolysaccharide of *Providencia stuartii*, and make the bacteria susceptible to serum killing.	exopolysaccharide	[86]
P560dep	Undefined	*K. pneumoniae*	phage P560	mice	inhibit biofilm formation	KL47 capsule polysaccharides	[62]
K2 depolymerase	Undefined	*A.baumannii*	vB_AbaP_B3	caterpillar larva and mice sepsis	Protects larvae and mice from *Acinetobacter baumannii* sepsis	capsular type K2	[87]
Dep42	Undefined	*K. pneumoniae*	SH-KP152226	In vitro	enhance polymyxin activity against *K. pneumoniae* biofilms	K47 capsule of *K. pneumoniae*	[15]
DepoKP36	Undefined	*K. pneumoniae*	vB_KpnS_KP36	Galleria mellonella	Degrade bacterial EPS	K63 capsule of *K. pneumoniae*	[13]
B9gp69	Undefined	*A.baumannii*	vB_AbaM_B9	In vitro	make K45 strain susceptible to serum killing	K45 and K30 capsule	[88]
B1dep	Undefined	*K. pneumoniae*	B1	In vitro	degrade the protective capsule on bacterial cells	K2 capsule	[89]
Dpo1	Undefined	*A. nosocomialis and A. baumannii*	Petty	In vitro	reduce viscosity, generate reducing ends in solution, indicative of hydrolase activity	capsular exopolysaccharides (EPS)	[90]
ORF96 protein	Undefined	*K. pneumoniae*	NTUH-K1790N	In vitro	decapsulation in the KN2 *K. pneumoniae* strains	KN2 capsule	[91]
Dpo42 and Dpo43	Undefined	*K. pneumoniae*	IME205	In vitro	Strip bacterial capsular polysaccharides, sensitize serum complement to kill host bacteria, have no haemolytic activity to erythrocytes	K47 capsule	[92]
S2–4, S1–1, S1–2, S1–3, S2–1, S2–2, S2–3, S2–6	Undefined	*Klebsiella*	ϕK64–1	In vitro	capsule depolymerase	K1, K11, KN4, K21, KN5, K25, K35, K64, K30, K69 capsule, respective	[93]
KN1dep, KN4dep, KN3dep and K56dep	Undefined	*Klebsiella*	KN1–1, KN4–1 and KN3–1	In vitro	capsule depolymerase	KN1, KN4, KN3 and K56 capsule, respective	[94]
K5–2 ORF37 and K5–2 ORF38	Undefined	*Klebsiella*	K5–2	In vitro	capsule depolymerase	K8, K30, K69, and K5	[95]
Dep622 and DepS8	Undefined	*K. pneumoniae*	vB_KpnP_Dlv622 and KpS8	Galleria mellonella larvae	Degrade polysaccharide and protect Galleria mellonella larvae from *K. pneumoniae* infection	K23	[66]

Sialidases (EC 3.2.1.18), also called neuraminidases, are a group of enzymes that hydrolyse the α-linkage of terminal sialic acids in glycans. Endosialidase E (endoE), derived from the *E. coli* K1 strain phage A192PP, selectively degrades sialic acid. However, endoE cannot kill pathogens or inhibit their growth, so its potential therapeutic efficacy remains unclear. Mushtaq *et al* [72,78]. conducted two studies to determine whether endoE could improve the outcome of *E. coli* K1 systemic infections in bacteraemia and meningitis rat models. The findings showed that the intraperitoneal administration of 20 µg of endoE can protected 3-day-old rats from systemic infection. The enzyme hydrolysed α-2,8-linked sialic acid and removed the capsular polysaccharides from the *E. coli* surfaces. Sensitization to the bactericidal effect of the complement system also occurred, and the phagocytic activity of the macrophages was enhanced when the capsular polysaccharides were removed.

Poly-γ-glutamate (γ-PGA) hydrolase (EC 3.4.19.9) is a type of peptidase that hydrolyzes γ-PGA into oligo-γ-glutamates. Kimura *et al* [73]. discovered a novel γ-glutamyl hydrolase PghP from *Bacillus subtilis* phage ΦNIT1, which randomly hydrolysed γ-PGA into oligo-γ-glutamates.

Hyaluronate (HA) lyases (4.2.99.1) and hyaluronidases (EC 4.2.2.1) are classes of enzymes that digest hyaluronate. Baker *et al* [80]. purified hylP-derived hyaluronidase from the *S. pyogenes* phage H4489A and reported that the phage HA lyase cleaved the N-acetylglucosaminidic bonds of hyaluronan and belonged to the category of hyaluronate lyases. The researchers further tested the substrate specificity and found that the phage HA lyase specifically cleaved hyaluronan, but not dermatan sulphate, keratan sulphate, chondroitin 4-sulphate, heparan sulphate, or heparin. This study may provide an alternative reagent to digest the *S. pyogenes* hyaluronan capsule. Another hyaluronate lyase named HylP1 from *Streptococcus pyogenes* prophage SF370.1 was tested to catalyse a β-elimination reaction of hyaluronan [79]. Although phage HA lyases and hyaluronidases play important roles in removing the streptococcal hyaluronan capsule, they can transform the nonvirulent strains into virulent strains [96].

Alginate lyases (EC 4.2.2) are a class of enzymes that catalyse the degradation of alginic acids, including mannuronate lyase (EC 4.2.2.3) and guluronate lyases (EC 4.2.2.11) [97]. To date, only *P. aeruginosa* and *A. vinelandii* phages are known to encode alginate lyases [97]. Glonti *et al* [81]. reported that *P. aeruginosa* phage PT-6 could rapidly reduce the viscosity of the alginic acid capsule by 62%–66% within 15 min. Furthermore, PT-6 alginase was purified from *A*. vinelandii phage suspensions. The enzyme corresponding to a 37 kDa band was shown to degrade polysaccharides to a series of oligouronides. By analysing these oligouronides and together with kinetic information, the authors concluded that PT-6 alginase exhibited polyuronide-degrading activity. The *A. vinelandii* phage is another example of a phage with alginate lyase activity [98].

Pectate lyases (EC 4.2.2.2) and pectin lyases (EC 4.2.2.10) are classes of pectolytic enzymes that degrade galacturonic acid. Gp17 is a pectate lyase obtained from the tail spikes of the *P. putida* phage φ15. Recombinantly expressed and purified Gp17 was reported to form opaque halo zones on the lawns of the host bacteria, which exhibited EPS-, CPS- or LPS-degrading properties [82]. Similarly, Liu *et al* [83]. reported that the phage IME200 exhibited polysaccharide depolymerase activity against *A. baumannii*. Based on the analysis of the complete genome sequences, open reading frame 48 was predicted to encode a polysaccharide depolymerase with pectate lyase activity (Dpo48), which was subsequently expressed, purified, and characterized. Dpo48 demonstrated high efficacy at a wide range of temperatures (20°C–70°C) and pH (5.0–9.0). It also exerted a synergistic effect with 50% serum on *A. baumannii* strains. Interestingly, another tail spike protein (TSP) with pectate lyase activity was derived from the *P. mirabilis* strain BB2000 phage (vB_PmiS_PM-CJR), which not only degraded the biofilms *in vitro* and reduced the adherence of bacteria to plastic pegs, but also improved the survival rates of *Galleria mellonella* larvae infected with the host cells [84].

Xylosidases (EC 3.2.1.37), levanases (EC 3.2.1.65), dextranases (EC 3.2.1.11), lipases (EC 3.1.1.3), and rhamnosidases (EC 3.2.1.40) catalyse the hydrolysis of xylan, levan, dextran, triacylglycerols, and rhamnogalacturonan, respectively. Some phages such as the *C. crescentus* transducing phage Cr30 [74], the *L. fermentum* phage phiPYB5 [75], the *B. subtilis* phage SP10 [76], and the *Shigella* phage SF6 [77] contain xylosidases, levanases, dextranases, lipases, respectively. These phage-derived enzymes are extremely rare [10], and have not been used for the treatment of bacterial biofilm until recently.

Apart from the defined classes of phage depolymerases, several undefined classes of phage depolymerases can inhibit the formation of bacterial biofilms or degrade those already formed. Shahed-Al-Mahmud *et al* [7]. reported that the TSP from the *A. baumannii* φAB6 phage inhibited the colonization of host cells on the surface of Foley catheters. The researchers also evaluated the therapeutic effect of TSP in zebrafish infected with *A. baumannii* 54149 and reported that the survival rate of zebrafish administered TSP (80%) was significantly higher than that of zebrafish administered PBS (10%). Dp49 is another capsule depolymerase identified from the *A. baumannii* phage vB_AbaM_IME285. Positivity for the depolymerase activity of Dp49 was found in 25 out of 49 *A. baumannii* clinical isolates [85]. In addition, Dp49 increased the serum killing ability against the strains Ab387 and Ab220 *in vitro*, whereas Dp49 the administration improved the survival rates of mice infected with Ab387 *in vivo*. Moreover, Oliveira et al. [86] identified a 604-amino-acid virion protein, gp52, with depolymerase activity. The tail spike gp52 purified from the *P. stuartii* phage vB_PstP_Stuart made the host bacteria susceptible to serum killing by degrading the exopolysaccharide. In another study, the *K. pneumoniae* phage P560 depolymerase P560dep was shown to inhibit biofilm formation. Intraperitoneal administration of a 50 μg dose of P560dep protected 90%–100% of mice infected with the KL47 carbapenem-resistant *K. pneumoniae* from mortality. Although the depolymerase was not related to the bacterial killing, the authors considered it an attractive and promising agent to combat infectious diseases [8]. The mechanisms by which parts of phage depolymerases degrade polysaccharides have been illustrated in certain studies focusing on the protein structures. A recent study by Squeglia *et al*. [70] revealed the crystal structure of the *Klebsiella* phage capsule depolymerase KP32gp38. It presented as a trimer in solution and in the crystal state. The monomer comprised four protein domains, a flexible N-terminal domain, a catalytic domain, a carbohydrate-binding domain, and a lectin-like fold C-terminal domain.

Phage depolymerase can also be used to treat Shiga toxin-producing *E. coli* (STEC) infections. The STEC strain HB10 O91 phage PHB19 was isolated, and the whole genome was sequenced and annotated by a phage study group. The authors [99] identified a novel phage depolymerase, Dep6, from the PHB19 TSP. *In vitro*, Dep6 effectively removed STEC biofilms and enhanced the susceptibility of host bacteria to serum killing. Furthermore, no toxic effects of Dep6 were observed in human red blood cells, lung carcinoma cells, or embryonic kidney cells *in vitro* and *in vivo*. In an STEC infection mouse model, pretreatment with Dep6 resulted in 100% survival compared with that in mice in the control group. Delayed treatment (3 h post infection) resulted in only 33% survival, whereas mice that were simultaneously treated with infection presented with a survival rate of 83%. In addition, the levels of proinflammatory cytokines, such as tumour necrosis factor-alpha, gamma interferon, and IL-1β, were reduced 24 h post infection in the Dep6-treated mice. However, the levels of IL-6 were not reduced. Thus, Dep6 appeared to be safe for mice based on the *in vivo* and *in vitro* assays. *P. aeruginosa* LPS can be degraded by phage depolymerase. LKA1gp49 is from *P. aeruginosa* phage LKA1 and cleaves β-band of LPS [100]. *K. pneumoniae* K63 capsule can also be degraded by phage depolymerase KP36gp50 [101].

These phage depolymerases possess the exopolysaccharide-degrading activity and glycoside hydrolases domains. One of the possible inhibitory mechanisms of a single phage depolymerase to bacterial biofilm may be the enzymes hydrolyse the glycosidic bonds of polysaccharide. There are four glycosidic bonds, including alpha-C-glycosidic bond, alpha-O-glycosidic bond, beta-C-glycosidic bond and beta-O-glycosidic bond. Each phage depolymerase may hydrolyse at least one kind of glycosidic bonds. However, phage depolymerases have specific and only one or a few types of capsular polysaccharide. We suspect that another possible inhibitory mechanisms may quench quorum sensing. Further studies will be done to explore whether phage depolymerases can disturb quorum sensing. And more phage depolymerases will be found to hydrolyse the glycosidic bonds of polysaccharide.

### Phage depolymerase cocktail application

The narrow specificity of phage depolymerases is one of the major obstacles in their use for removing bacterial biofilms and has considerably restricted their application. Several studies have attempted to broaden the specificity via protein engineering and combining several different phage depolymerases to form cocktails (Table 2).

Few phage depolymerases have been tested in terms of their ability to degrade different types of bacterial capsules *in vivo*. Whether phage depolymerases can exhibit generalized therapeutic efficacy towards different kinds of capsules has remained unclear. For example, more than 80 different types of capsules were discovered based on the serological, biochemical, and genetic properties of *E. coli* [102]. Lin *et al* [63]. used five phage depolymerases to combat three capsule types in mouse infection models. The phage depolymerases K1E, K1F, K1H, K5, and K30 (gp41 and gp42) were cloned and purified *in vitro*. In a mouse thigh model, the majority of the mice were rescued by treatment with all of the phage depolymerases, except K1E, when the enzyme (20 µg per mouse) was injected within 0.5 h after the bacterial injection. Preliminary trials showed that the effective doses of K1F and K1H ranged between 2 and 5 µg per mouse. The effective dose of K5 ranged between 2 and 20 µg per mouse, whereas the effective dose of K30 gp41 was 20 µg per mouse. K30 gp42 did not appear to improve the survival rate, and K30 gp41 was found to be less effective than K1F, K1H, and K5. At a dose of 20 µg per mouse, the mixture of K30 gp41 and K30 gp42 rescued all three mice (3/3), whereas K30 gp41 rescued three out of eleven (3/11) mice, and K30 gp42 did not rescue any of the mice (0/3). Although the survival outcome appeared to be somewhat better in the mixture group than that in the two individually treated groups, the sample size was too small to draw any definitive conclusions. The potential acute toxicity of the five phage depolymerases (K1E, K1F, K1H, K5, and K30 gp41) was evaluated by injecting 100 µg of the different phage depolymerases into the right thigh and monitoring the survival, body weight gain, and behaviour of the mice. All of the mice appeared healthy without any change in behaviour over an observation period of 5 days. Statistical analysis indicated no significant differences in body weight gain between the phage depolymerase- and PBS-treated mouse groups. Nonetheless, further studies are required to confirm the safety and efficacy of applying phage depolymerase cocktails.

A cocktail of some phage depolymerases can be used to treat infection with *K. pneumoniae*. Recently, Pan *et al* [93]. reported that the multi-host *Klebsiella* phage ϕK64–1 can infect the capsules of *Klebsiella* K1, K11, KN4, K21, KN5, K25, K35, K64, K30, and K69. To analyse the wide capsular-type – specific host spectrum, the authors expressed and purified putative proteins (S2–4, S1–1, S1–2, S1–3, S2–1, S2–2, S2–3, S2–5, and S2–6) that exhibited similarity with tail fiber/spike or lyase proteins. In terms of capsule depolymerization activities of these proteins, opaque halo zones were observed using a through spot test of capsule depolymerase on the individual bacterial lawns of K1, K11, KN4, K21, KN5, K25, K35, K64, and K30/K69. S2–6 could digest the capsules of both type K30 and type K69 bacteria. In another study, the same group detected other *Klebsiella* phage depolymerases, such as KN1dep, KN4dep, KN3dep, and K56dep, with high specificities for capsular serotypes KN1, KN4, KN3, and K56, respectively [94]. Moreover, Majkowska-Skrobek *et al* [103]. discovered a novel *Klebsiella* phage KP32, which produced two capsule depolymerases, KP32gp37 and KP32gp38, and infected the K3 and K21 capsular serotypes, respectively. Both depolymerases increased the lifespan of the *Galleria mellonella* larvae, which were infected with *K. pneumoniae* in a time-dependent manner. In another study, Liu *et al* [92]. identified a new *K. pneumoniae* phage IME205 with two phage depolymerases (Dpo42 and Dpo43), which were stable across a relatively broad temperature range (20°C–70°C). The pH levels of Dpo42 and Dpo43 were in the ranges of ranged between 5.0–8.0 and 4.0–8.0, respectively. The two polymerases could strip the K47 CPSs and sensitize the serum complement to kill the host bacteria but did not exhibit any haemolytic activity against erythrocytes. Thus, Dpo42 and Dpo43 could be used to combat the K47 capsule in *K. pneumoniae* infections. Moreover, *Klebsiella* phage K5–2 was found to encode two capsule depolymerases K5–2 ORF37 and K5–2 ORF38. K5–2 ORF37 exhibited K8, K30, and K69 depolymerase activities, whereas K5–2 ORF38 exhibited K5 depolymerase activity [95]. Owing the clear capsular types of *K. pneumoniae* that phage depolymerases can degrade, phage depolymerases have the potential to combat different capsular types when used as a cocktail.

Although a cocktail can broaden the antibacterial biofilm spectrum, no studies have focused on the different phage depolymerases used in cocktails to combat the bacteria capsules. Phage cocktail therapy has been used to treat *M. abscessus* infection [104], *P. aeruginosa* respiratory infection [105], and *E. coli* urinary tract infection in a mouse model [106]. In addition, some phage cocktails have been used in clinical trials, such as *P. aeruginosa* phage cocktail to treat burn wound infection (phase 1/2 trial) [107], oral *E. coli* phage cocktail to treat acute bacterial diarrhoea [108], and topical *C. acnes* phage cocktail to treat skin acne (phase 1 trial) [109], have been used in clinical trials. Additional studies are required to explore the effect and safety of phage depolymerase cocktails.

Phage depolymerase cocktail application mainly use the mechanism and effect of single depolymerase. Due to the high specificity, a single phage depolymerase can degrade only one or a few types of capsular polysaccharide. This kind of single phage depolymerase application limits the antibiofilm spectrum. In order to enlarge the antibiofilm spectrum, phage depolymerase cocktail application is one of the solutions. Two or more different phage depolymerase may hydrolyse different kinds of glycosidic bonds of polysaccharide. This cocktail application can improve the antibiofilm effect. In addition, phage depolymerase cocktail application can also deduce the resistance issue. Although the probability of polysaccharide mutation is low, the resistant mutation may generate when phage and the host (pathogenic bacteria) have coevolved over time.

### Combination therapy with phage depolymerase and antibiotics

Although a single phage depolymerase tends to be somewhat effective in controlling bacterial biofilms, the high specificity of depolymerases limits the complete removal of the biofilm owing to variations in the EPS. In some cases, phage depolymerases might exert anti-polysaccharide activity against a small set of bacterial strains. To address the limitation of the narrow host spectrum, combination therapy with phage depolymerases and antibiotics, an approach widely adopted in humans, may prove useful in combating infections caused by biofilm-forming pathogens. The degradation of the biofilm matrix by phage depolymerases can increase the penetration of antibiotics to exert synergistic effects (Table 3).

**Table 3.** Combination therapy of phage depolymerase and some antibiotics in the treatment of bacterial biofilm.

Depolymerase	Origin phage	Phage family	Bacterium genus	Antibiotics	Combined outcome	Substrate	Application model	Study references
Dep42	SH-KP152226	Podoviridae	*K. pneumoniae*	polymyxin	synergy	K47 capsule	In vitro	[15]
KPO1K2 associated depolymerase	KPO1K2	-	*K. pneumoniae*	ciprofloxacin	synergy	biofilm	In vitro	[110]
DepoKP36	vB_KpnS_KP36	Siphoviridae	*K. pneumoniae*	ciprofloxacin, oxytetracycline, and chloramphenicol	synergy	K63 capsule	Galleria mellonella	[13]
Dpo71	IME-AB2	-	*A. baumannii*	colistin	synergy	Biofilm	Galleria mellonella	[16]
KP34p57	KP34	-	*K. pneumoniae*	ciprofloxacin	Neutral	Biofilm	In vitro	[111]
DpoMK34	vB_AbaP_PMK34	-	*A. baumannii*	Imipenem, amikacin, and colistin	Neutral	K2 capsule	In vitro	[14]
TF	φAB2	-	*A. baumannii*	colistin	antagonism	exopolysaccharide (EPS)	In vitro	[112]

Ciprofloxacin is one of the most commonly used antibiotics in clinic [113]. Some phage capsule depolymerases exhibit synergy with ciprofloxacin, whereas others demonstrate no synergistic effects with ciprofloxacin. Verma *et al* [110]. discovered a depolymerase derived from *K. pneumoniae* phage KPO1K2. The depolymerase and ciprofloxacin could reduce the bacterial numbers in mature 3-day (72 h) biofilms. The antibiofilm effect was reported to be significantly greater in the combination treatment group than in the group treated with ciprofloxacin alone (*P* > 0.05). Interestingly, the concomitant application of depolymerase and ciprofloxacin produced different effects on the biofilm. When applied concomitantly for 6 h, an insignificant reduction of 1.21 + 0.62 logs was observed in the biofilm bacterial count. However, when treated with depolymerase for 60 min followed by 6 h treatment with ciprofloxacin, a significant reduction of 3.72 + 1.2 logs was observed in the bacterial count. These findings indicate that the combined treatment with phage depolymerase and ciprofloxacin is effective in mature biofilms. However, some studies have reported contradictory findings. For example, Latka and Drulis-Kawa [111] identified a *K. pneumoniae* phage depolymerase called KP34p57, which had no impact on ciprofloxacin activity. Their findings showed that the The combination of KP34p57 and ciprofloxacin did not improve the antibiotic activity. Moreover, the phage KP36 capsule depolymerase DepoKP36 did not affect the susceptibility of biofilm-forming *K. pneumoniae* strains to antibiotics such as ciprofloxacin, oxytetracycline, and chloramphenicol [13].

Colistin is the last-resort antibiotic for clinical multidrug-resistant Gram-negative bacterial infections. The combination of phage depolymerase and colistin may produce a synergistic effect to combat *A. baumannii* infections. Chen *et al* [16]. expressed a novel depolymerase Dpo71 from the *A. baumannii* phage IME-AB2 *in vitro*, which inhibited biofilm formation and interfered with the preformed biofilm. In addition, Dpo71 enhanced the bactericidal effect of colistin. Single-dose colistin (1 µg/mL, 1/2 MIC) alone or with 5% serum did not influence its antibacterial effect against the host cells. However, the application of 10 µg/mL Dpo71 + 1 µg/mL of colistin along with 5% serum resulted in a significant reduction in the counts of *A. baumannii*. This combination treatment resulted in a 7-log reduction in the bacterial count, and the antibacterial effect was boosted to nearly complete eradication. In comparison to 1 µg/mL colistin alone and 10 µg/mL dpo71 alone treatment, bacterial reduction was a 1-log and 0-log, respectively. The authors also revealed the underlying mechanisms for the superior action of the combination therapy compared with the individual treatments. Scanning electron microscopy showed that the bacterial capsule was stripped by Dpo71 and that the host cell surface did not contain any pilus-shaped protrusions. In contrast, the surfaces of the bacterial cells in the untreated group presented with both capsules and pilus-shaped protrusions. The removal of the capsule by phage depolymerase Dpo71 significantly enhanced the outer membrane destabilization ability of colistin, which promoted the interaction between antibiotics and the bacteria and facilitated the entry of the drug into the bacterial host. In infection models, the combination of Dpo71 and colistin could improve the survival rate of *A. baumannii*-infected *Galleria mellonella*. Although Dpo71 itself had no bactericidal efficacy, treatment with Dpo71 alone rescued 40% of the infected *Galleria mellonella* over a period of 72 h. However, the combined treatment of Dpo71 and colistin rescued 80% of the infected worms during the same observation period. Approximately 70% of the infected worms died within 18 h, and the mortality rate increased to 90% after 48 h. These results indicate that phage depolymerases can act as adjuvants with some antibiotics to enhance antibacterial activity. Nevertheless, the combination of phage depolymerase and colistin may not produce a synergistic effect. Luo *et al* [112]. identified another *A. baumannii* phage depolymerase called TF, which did not exhibit any additive or synergistic effects with colistin on the host bacteria. Surprisingly, a temporary increase in the resistance of *A. baumannii* to colistin was observed after the EPS was peeled by phage depolymerase TF from the bacteria. Thus, it was considered that the loss of EPS may have reduced the colistin attachment, causing a temporary increase in resistance.

Polymyxin has been shown to exert the synergistic effects with phage depolymerase. *K. pneumoniae* phage SH-KP152226 depolymerase Dep42 increased the antibacterial activity of polymyxin when they were used together [15]. The average bacterial count of the Dep42 + polymyxin treatment group was 5.260 ± 0.05 log, whereas those of the groups treated with Dep42 or polymyxin alone were 6.317 ± 0.01 and 6.013 ± 0.125 log, respectively, which demonstrates a significant reduction of 0.743 ± 0.05 log compared with that in the polymyxin group. Collectively, the findings of these studies strongly indicate that phage depolymerase Dep42 and polymyxin have a synergistic effect on multidrug-resistant *K. pneumoniae*.

Some antibiotics is neither synergistic nor antagonistic in combination with phage depolymerases. *A. baumannii* MK34 phage vB_AbaP_PMK34 capsule depolymerase DpoMK34 was neither synergistic nor antagonistic in combination with different antibiotics, such as colistin, imipenem, and amikacin [14]. However, the addition of 500 µg/mL of DpoMK34 did not change the MIC values of the three tested antibiotics upon pretreatment or cotreatment with MK34 and DpoMK34. It was, thus, the authors concluded that DpoMK34 did not produce synergistic or antagonistic effects in combination with colistin, imipenem, and amikacin.

Some antibiotics, such as ciprofloxacin, colistin, polymyxin, have a synergistic effect with a certain phage depolymerase. While some antibiotics, such as imipenem, amikacin, have no synergistic effect with phage depolymerases. The most likely reason for the synergistic effects of phage depolymerases and some antibiotics is that the removal of CPS by phage depolymerases helps some antibiotics to access the bacterial surface. This changes the arrangement of bacteria in the biofilm to a dispersed state, which enhances the ability of antibiotics to penetrate into the biofilm. Eventually, the intensity of the antimicrobial attack is enhanced with the help of the phage depolymerase.

### Phage depolymerase enhances the effects of human and mouse immune systems

CPSs are important virulence factors that help bacteria to escape the human immune system. Surface polysaccharides provide shields against components of the host immune system, such as the complement system and phagocytosis. Loss or alteration of CPSs can make bacteria more susceptible to clearance by the human immune system. Table 4 lists some studies on how phage depolymerases enhance the susceptibility to human serum and kill bacteria.

**Table 4.** Phage depolymerases enhance the effect of human immune system in the treatment of bacterial biofilm.

Depolymerase	Origin phage	Phage family	Bacterium genus	Final concentration of depolymerase	bacteria reduction number/log	Substrate	Application model	Study references
K2 depolymerase	vB_AbaP_B3	-	*A. baumannii*	50 µg/l	4	K2 capsule	caterpillar larva and mice sepsis	[87]
DpoMK34	vB_AbaP_PMK34	-	*A. baumannii*	100 µg/ml	5.05	K2 capsule	In vitro	[14]
Dpo48	phage IME200	-	*A. baumannii*	10 µg/ml	5	galacturonic acid	In vitro	[83]
B9gp69	vB_AbaM_B9	Myoviridae	*A. baumannii*	0.1 µM	4.5	K45 and K30 capsule	In vitro	[88]
Dep-ORF8	PHB02	Caudovirales	*Pasteurella multocida*	100 µg/ml	3.5–4.5	serogroup A capsule	mice	[114]
KP32gp37	KP32	-	*K. pneumoniae*	0.1 µg/ml	1.6 log upon 3 h exposure and almost 4 log upon 7 h exposure	K3 capsule	Galleria mellonella larvae	[103]
KP32gp38	KP32 [88]	-	*K. pneumoniae*	100 µg/ml	1.7–4.6 log upon 3 h exposure	K21 capsule	Galleria mellonella larvae	[103]
Dep6	PHB19	Autographivirinae	Shiga toxin-producing *Escherichia coli* (STEC)	30 µg/m	4.2	lipopolysaccharide (O91)	mice	[99]
DP	IME180	Podoviridae	*Pseudomonas aeruginosa 1193*	25 μg/ml	2	exopolysaccharides of *Pa*.1193	In vitro	[115]

Phage-derived capsule depolymerases can remove bacterial CPSs, and some phage depolymerases are reported to make the pathogens susceptible to serum killing. In recent years, *A. baumannii* has been considered to be an important nosocomial pathogen in intensive care units [116]. Most clinical isolates are resistant to all available antibiotics and the current treatment methods are becoming less effective. One reason for this resistance is that the CPSs protect the bacteria from antibiotics via the polysaccharide structure and aid in their evasion of the host immune system. In other words, CPSs are a major virulence factor. Oliveira *et al*. [87] purified a K2 capsule-specific depolymerase from the tail spike C-terminus of the *A. baumannii* phage vB_AbaP_B3 and assessed its activity *in vivo*. It was found to protect caterpillar larva and mice against bacterial infections. In a mouse sepsis model, the intraperitoneal injection of K2 depolymerase (dose, 50 μg) resulted in 60% of mice avoiding mortality due to infection. Additionally, significant reductions in the expression levels of tumour necrosis factor-alpha and interleukin-6 were observed. K2 depolymerase enhanced the ability of human serum to kill the host cells and reduced the number of bacteria to < 10 colony-forming units (CFU)/mL. The NIPH 2061 strain was not susceptible to serum killing following the inactivation of K2 depolymerase. It was, thus, concluded that the human complement system was activated to control the infection via the action of K2 depolymerase, which degraded the CPS and, consequently, affected the bacterial virulence.

DpoMK34 is another *A. baumannii* phage depolymerase, which was reported to increase the ability of the serum to kill *A. baumannii* MK34 in a concentration-dependent manner [14]. Cells treated with 100 µg/mL DpoMK34 presented with a 1.8 ± 0.34 log reduction in 25% (v/v) human serum and a 5.05 log unit reduction in both 50% (v/v) and 75% (v/v) human sera when compared with that in PBS-treated cells. Moreover, heat inactivation of DpoMK34 did not cause any reductions in bacterial cell numbers, even in the 75% (v/v) human serum. Similar findings were reported in another study, wherein a 5 log reduction in Dpo48-treated bacteria was observed following treatment with a 50% volume of serum. The inactivation of the complement in the serum did not result in any reduction in the serum-dependent bacterial count [83]. Oliveira *et al* [88]. identified another *A. baumannii* phage depolymerase called B9gp69, which rendered K45 strains susceptible to serum killing *in vitro*. The number of depolymerase-pretreated bacteria incubated with serum was reduced to below the detection limit (<10 CFU/mL). Notably, B9gp69 digested the capsule polysaccharides of both K30 and K45 strains. Furthermore, the optimal activity of the enzyme could be maintained at temperatures ranging from 20°C to 80°C and pH values ranging from 5 to 9. These are clear indications of the effect of phage depolymerase on the bacterial susceptibility of bacteria to human serum killing.

The use of other phage depolymerases, such as Dep-ORF8 from *P. multocida* phage PHB02, which specifically degrades the serogroup A capsule, has been reported. The purified Dep-ORF8 significantly increased the survival of mice infected with *P. multocida*. Additionally, it did not increase the eosinophil and basophil counts or cause any other pathological changes when compared with the control group. Human serum, mouse serum, and mouse whole blood alone exhibited minor bactericidal effects of 1.2–1.7 log CFU reductions in *P. multocida* strain HB03 cell counts. Treatment with Dep-ORF8 + serum further reduced the cell counts (by 3.5–4.5 log CFU). However, no significant difference in viable cell counts was observed between the Dep-ORF8 + whole blood and the Dep-ORF8 + serum treatment groups. Heat inactivation of the serum resulted in an increase in the survival counts of the bacterial cells to levels equal to those in the PBS control group [114].

- *pneumoniae* phage depolymerases can sensitize cells to serum complement-mediated killing. KP32gp37 and KP32gp38 obtained from *Klebsiella* phage KP32 increased the sensitivity of serum-resistant cells to complement-mediated host bacterial killing. Decapsulation of the strains by depolymerases resulted in the exposure of the ligands to phagocytic cell attachment [103]. Thus, phage depolymerases could combat the resistance of biofilm bacteria and increase the phagocytic activity of the cells, thereby killing the biofilm bacteria. KP32gp37 increased the phagocytic cell uptake of strain 271 by approximately two folds. Similarly, the geometric mean fluorescence intensity (gMFI) value of the KP32gp38-treated strain 358 (146.7 ± 13.6 gMFI) was higher than that of the untreated bacteria (83.9 ± 5.7 gMFI). In the case of strain 968, the gMFI value in the KP32gp38-treated group (102.3 ± 6.8) was lower than that in the untreated group (293.6 ± 24.4). Evaluation of the time-dependent bactericidal effect of depolymerases + serum against *K. pneumoniae* strains 271, 45, and 358 revealed that 0.1 µg/mL of KP32gp37 could lead to a 1.6 log decrease in the bacterial number after 3 h of exposure and a 4 log decrease after 7 h of exposure in the case of strain 271. For the K21-type strains, including strains 45 and 358, 100 µg/mL of KP32gp38 caused only a 1.7–4.6 log reduction in the bacterial number compared with that in the initial inoculum after 3 h of exposure. After 7 h of exposure, only minor reductions were observed in the depolymerase-treated *Klebsiella* strains 358 and 45 [103]. Similarly, Dep6 derived from the STEC phage PHB19 TSP enhanced the serum sensitivity of the host strain. Dep6 at 30 µg/mL resulted in a 4.2 log reduction in the number of host bacteria, whereas that in the PBS-treated group remained at 8 × 10^8^ CFU/mL [99].
- *aeruginosa* phage depolymerase DP is another example of enhancing bactericidal activity mediated by serum in vitro [115]. The authors have isolated a lytic *P. aeruginosa* phage named IME180 from the sewage of a hospital. Through genomic sequence analysis of IME180 phage genome, DP has two catalytic regions, the Pectate lyase_3 super family and Glycosyl hydrolase_28 super family. The phage depolymerase DP can degrade exopolysaccharide of *P. aeruginosa* and enhance serum bactericidal activity in vitro. In the bactericidal assay, the bacterial enumeration reduces by two orders of magnitude in the serum and DP group. However, when either serum or DP is applied to bacteria individually, the bacterial enumeration is no obvious decrease or increase. In other words, the *P. aeruginosa* phage depolymerase DP has the potential to be an anti-microbial agent targeting *P. aeruginosa*.

The human immune system can not recognize pathogens when bacteria generate CPSs and encase themselves. CPSs help bacteria to escape the human immune system and bacteria fail to induce human immune response. When phage depolymerases strip the protective polysaccharide layers from the pathogen cells, which exposes them to the immune system. And the uncapsulate bacteria expose the lipoteichoic acids of the Gram-positive bacterial cell wall, lipopolysaccharide and outer membrane protein of the Gram-negative bacterial outer membrane. These structural component of the bacterial cell wall induce and activate the immune response, especially the complement system and macrophagocytes.

## Conclusions

The resistance of bacterial biofilms to antibiotics and the human immune system prompted scientists to search for alternative methods to counter antibiotic-resistant strains. Studies have characterized several phage depolymerases that act against biofilm-forming bacteria, such as *K. pneumoniae*, *E. coli*, *A. baumannii*, *P. mirabilis*, *P. aeruginosa*, *S. pyogenes*, *B. subtilis*, and *P. stuartii*. As natural antimicrobial agents, phage depolymerases clearly have a potential role in preventing and treating bacterial biofilm-associated infections. Although phage depolymerases have been demonstrated to possess potential antibiofilm activity *in vitro*, no clinical trials on this approach have so far been conducted thus far. There is, thus, a need for detailed exploration of the use of phage depolymerases as potential therapeutic drugs. Fortunately, some phage endolysins have entered clinical trials. Considering the current biosafety standards and regulations, the clinical application of phage depolymerases is much easier than that of the phage itself. Nonetheless, there is a need for further studies confirming the therapeutic use of phage depolymerases in humans. Besides, the use of phage depolymerases in combinations with some innovative treatments such as antibacterial peptide, nano particle, silver, copper and zinc will be explored in the future. As it acts as a kind of enzymes, phage depolymerase can be synthesized with nanomaterials to form phage depolymerase-incorporated nanomaterials. Or liposome-coupled phage depolymerases were prepared. Then, the potential therapeutic effect of innovative treatments will be evaluated in future.

## Data Availability

Data sharing is not applicable to this article as no new data were created or analysed in this study.
